# Antihelix ulcer in anti-MDA5-positive dermatomyositis

**DOI:** 10.1093/rap/rkaf117

**Published:** 2025-10-09

**Authors:** Wenhan Huang, Lin Tang

**Affiliations:** Department of Rheumatology and Immunology, The Second Affiliated Hospital of Chongqing Medical University, Chongqing, China; Department of Rheumatology and Immunology, The Second Affiliated Hospital of Chongqing Medical University, Chongqing, China

A 44-year-old woman presented to the rheumatology clinic with a 2-month history of cutaneous eruption, arthralgia and fatigue. Physical examination revealed Gottron’s papules, mechanic’s hands, V-neck sign and antihelix erythema with ulcer formation (Fig. 1). Proximal muscle strength was 4 according to the Medical Research Council scale. Laboratory findings revealed elevated ESR (52 mm/h) and serum ferritin (991.4 ng/ml). The level of creatine kinase was 487 U/l (reference range: 38–174). Test for anti-melanoma differentiation-associated protein 5 (MDA5) antibodies and anti-Ro52 antibodies were positive. Chest CT showed interstitial lung disease. MRI showed a high signal of thigh muscles on the T2-weighted image. The diagnosis of anti-MDA5-positive dermatomyositis was made. Treatment with methylprednisolone (i.v. therapy, 80 mg/d), cyclophosphamide (i.v. therapy, 0.4 g once/week) and tacrolimus (oral, 1 mg twice a day; the blood trough concentration was maintained at 5–10 ng/ml) was initiated.

**Figure:**
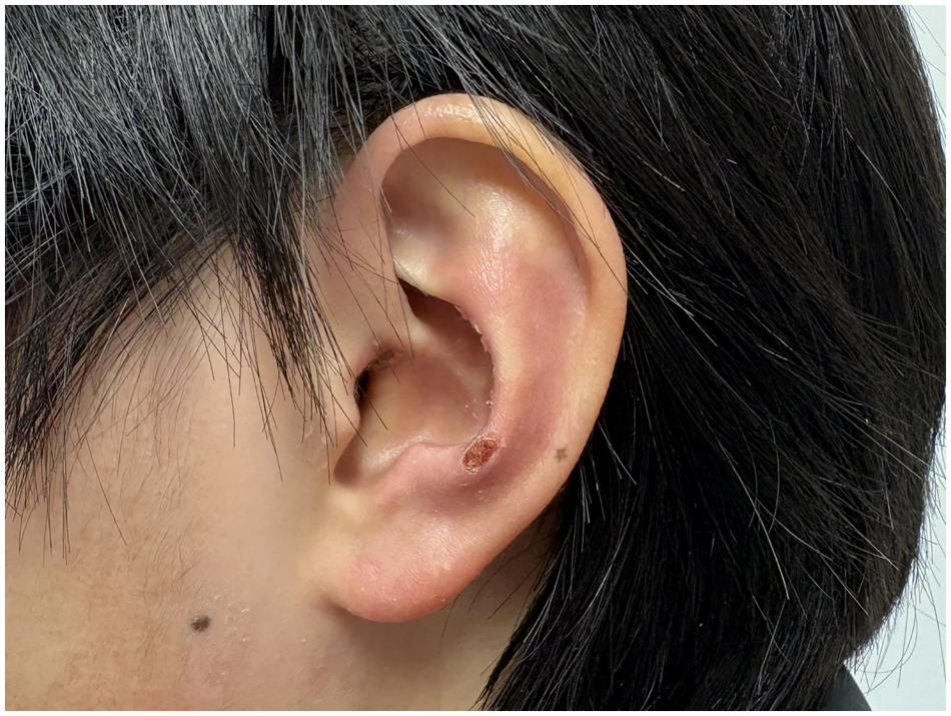
**Figure**  **1.** Antihelix erythema with ulcer formation

Interstitial lung disease and skin involvement were two significant features of anti-MDA5 dermatomyositis. Apart from common manifestations, such as Gottron’s papules, V-neck and shawl sign, skin lesions in other areas were gradually being recognized as well. Antihelix and helix erythema were relatively rare in anti-MDA5 dermatomyositis. On the one hand, it is regarded as microvascular injury induced by pressure, just like decubitus [1]. On the other hand, it has been reported that persistent endoplasmic reticulum stress and unfolded protein response induced by the combination of MDA5 protein and protein kinase RNA-like endoplasmic reticulum kinase might be an important mechanism of vascular damage in anti-MDA5 dermatomyositis patients [2]. This patient has developed antihelix ulcers, indicating more severe vasculitis. Early combined immunotherapy is helpful to control the development of anti-MDA5 dermatomyositis.

## Data Availability

The data underlying this article are available in the article.
